# Production and separation of ^163^Ho for nuclear physics experiments

**DOI:** 10.1371/journal.pone.0200910

**Published:** 2018-08-22

**Authors:** S. Heinitz, N. Kivel, D. Schumann, U. Köster, M. Balata, M. Biasotti, V. Ceriale, M. De Gerone, M. Faverzani, E. Ferri, G. Gallucci, F. Gatti, A. Giachero, S. Nisi, A. Nucciotti, A. Orlando, G. Pessina, A. Puiu, S. Ragazzi

**Affiliations:** 1 Nuclear Energy and Safety Department, Paul Scherrer Institut, Villigen, Switzerland; 2 Institut Laue-Langevin, Grenoble, France; 3 Laboratori Nazionali del Gran Sasso, Assergi, Italy; 4 Dipartimento di Fisica, Universita di Genova, Genova, Italy; 5 Istituto Nazionale di Fisica Nucleare Genova, Genova, Italy; 6 Dipartimento di Fisica, Universita di Milano-Bicocca, Milano, Italy; 7 Istituto Nazionale de Fisica Nucleare, Milano, Italy; Los Alamos National Laboratory, UNITED STATES

## Abstract

This paper describes the production and chemical separation of the ^163^Ho isotope that will be used in several nuclear physics experiments aiming at measuring the neutrino mass as well as the neutron cross section of the ^163^Ho isotope. For this purpose, several batches of enriched ^162^Er have been irradiated at the Institut Laue-Langevin high flux reactor to finally produce 6 mg or 100 MBq of the desired ^163^Ho isotope. A portion of the Er/Ho mixture is then subjected to a sophisticated chemical separation involving ion exchange chromatography to isolate the Ho product from the Er target material. Before irradiation, a thorough analysis of the impurity content was performed and its implication on the produced nuclide inventory will be discussed.

## Introduction

The radioactive isotope ^163^Ho (t_1/2_ = 4567 a [1,2]) has gained considerable attention within the physics community due to its very low Q-value for electron capture decay of only 2.8 keV [3,4]. This property and recent developments in high precision calorimetric measurements have facilitated several research projects devoted to measuring the mass of the neutrino. Among them, the HOLMES collaboration aims at implanting 3x10^5^ Bq of isotopically pure ^163^Ho a grid of 10^3^ transition edge sensor micro-calorimeters to precisely measure the end-point region of the energy spectrum of the ^163^Ho electron capture decay [5]. With an envisaged statistical mass sensitivity around 1 eV, this measurement will provide an alternative technique to spectrometry to answer the long lasting question in physics about the neutrino mass [6].

The isotope ^163^Ho is also an interesting nuclide in terms of nuclear physics research in the field of bound state beta decay. It has been experimentally observed that under fully ionized conditions (such as in stellar environments), the previously stable ^163^Dy isotope becomes radioactive and decays to ^163^Ho with a half-life of 47 d [7]. This circumstance significantly influences the s-process pathway in the A = 163 mass region, opening an additional branch towards isotopes accessible mainly via the p-process. Within this context, the neutron-capture cross section of the ^163^Ho isotope is foreseen to be measured at the n_TOF CERN facility at thermal to stellar energies for the first time. While the Maxwellian average neutron capture cross-section (MACS) of ^163^Ho has been measured in 1995 at stellar energies [8], a new measurement with several mg quantities of ^163^Ho is envisaged to determine the neutron capture cross section in the eV to keV region typical for stellar environments [9].

**Fig 1:**
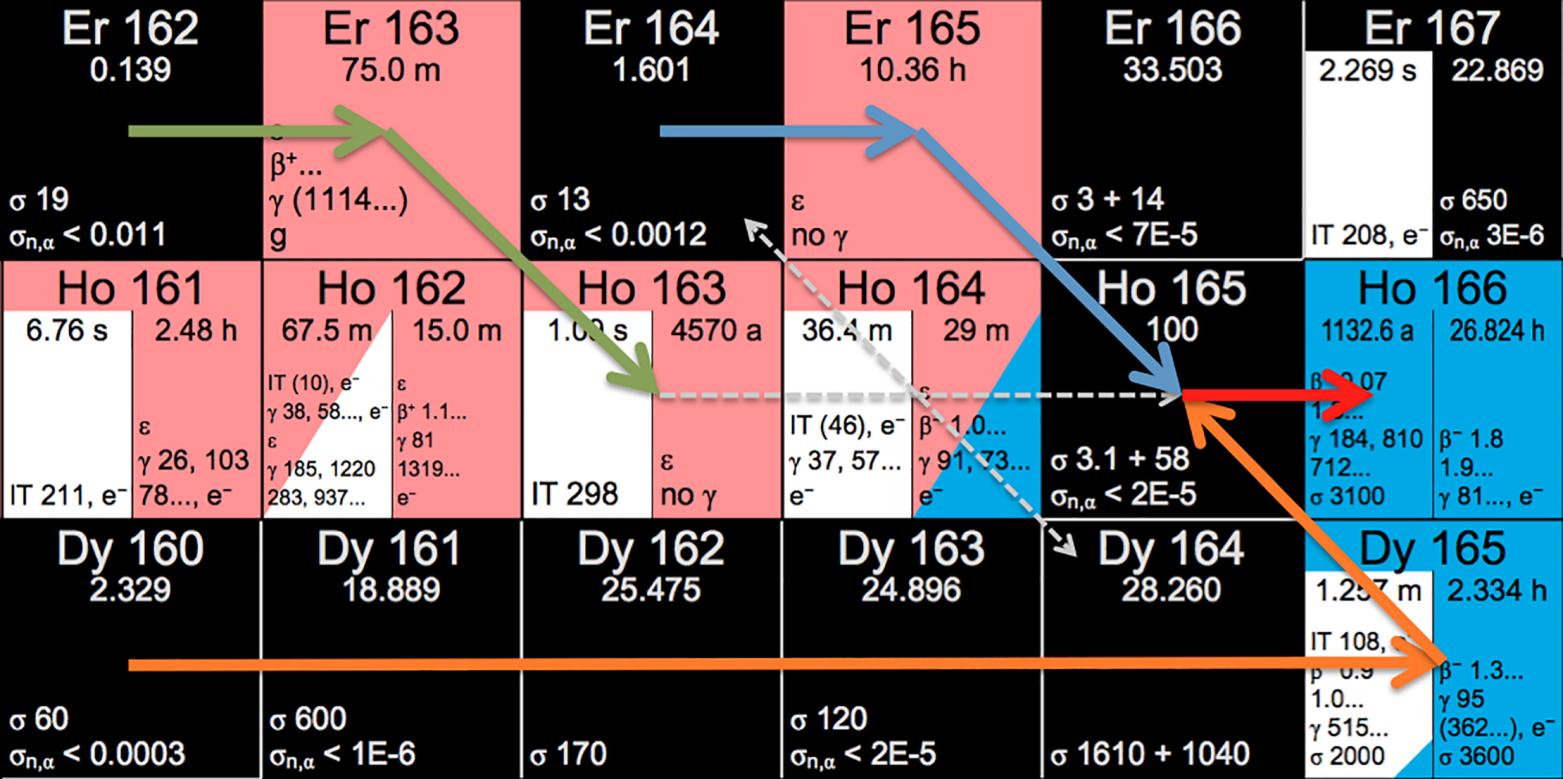
Excerpt from the nuclide chart showing the main production route of ^163^Ho (green arrows) together with parasitic formation of ^166m^Ho (red arrow) from ^165^Ho. The latter is formed by neutron captures from ^164^Er (blue arrows) or from Dy isotopes (orange arrows) within the irradiated material. Reprinted from nucleonica.com / Karlsruhe Nuclide Chart Online under a CC BY license, with permission from Dr. Joseph Magill, original copyright 10^th^ Edition, 2018.

In collaboration with the Institut Laue-Langevin (ILL), Grenoble, France, and the Paul Scherrer Institut (PSI), Villigen, Switzerland, the HOLMES project aims at producing roughly 100 MBq of ^163^Ho by irradiation of enriched ^162^Er material in a nuclear reactor. The production route ^162^Er(n,γ)^163^Er followed by decay to ^163^Ho is depicted in Fig 1 together with other neutron capture reactions. While neutron irradiation of ^162^Er has been shown to be the most efficient way of producing the ^163^Ho isotope [10,11], alternative routes via proton irradiation of ^nat^Dy or enriched ^164^Dy foils have also been reported in literature [1,12,13]. The HOLMES collaboration has chosen to pursue the former production method due to higher ^163^Ho production yields [10] and better availability of irradiation resources, taking into account its unavoidable disadvantage.

The main drawback arising from neutron irradiation of enriched Er is the inevitable formation of ^166m^Ho (see Fig 1). This isotope has a half-life (t_1/2_ = 1132 a [14]) comparable to that of ^163^Ho and has a complex decay scheme, which drastically deteriorates calorimetric decay measurements of ^163^Ho contaminated with ^166m^Ho [5]. Since the latter isotope cannot be separated from ^163^Ho by chemical means, a mass separation of these two isotopes is mandatory before the implantation of Ho into a calorimetric measurement system [5]. The main production pathways of ^166m^Ho may be summarized as follows:

1. from neutron captures on ^165^Ho present as impurity in the irradiated Er material;
2. from the reaction ^164^Er(n,γ)^165^Er(EC) towards ^165^Ho and subsequent reaction 1;
3. from capture reactions of ^164-x^Dy(xn,γ)^165^Dy(β^-^) towards ^165^Ho and subsequent reaction 1;
4. from neutron captures on ^163^Ho to ^164g+m^Ho, that undergo either EC decay feeding reaction 3 or β^-^ decay towards ^164^Er feeding reaction 2.

**Fig 2:**
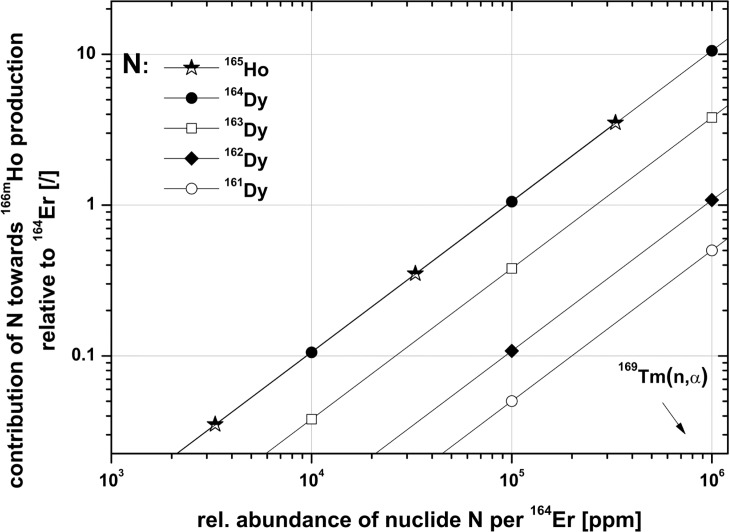
Contribution towards the production of ^166m^Ho from Ho and Dy isotopes as function of their concentration relative to ^164^Er. Calculated for a 50 d irradiation in a thermal neutron flux of 10^15^ n cm^-2^ s^-1^. Calculations were performed using the ChainSolver code [20]. Ho and ^164^Dy come to lie nearly on the same line since under the given flux ^164^Dy proceeds via reaction 3 almost completely to ^166m^Ho, only a minor fraction leads via double capture reactions to ^166^Dy(β^-^)^166g^Ho.

The production rates of ^166m^Ho from ^165^Ho and the isotopes ^161^Dy, ^162^Dy, ^163^Dy and ^164^Dy as function of their concentration relative to ^164^Er for a 50 d irradiation with a thermal neutron flux of 10^15^ n cm^-2^ s^-1^ are given in Fig 2. As can be seen, the dominant impurities transmuting into ^166m^Ho are ^165^Ho and ^164^Dy. While the main production pathway surely proceeds over the ^164^Er(n,γ) reaction, an impurity content of 5% relative to ^164^Er of ^165^Ho or ^164^Dy almost equally contributes to the ^166m^Ho production. Since the thermal neutron capture cross section of ^166m^Ho is in the order of 3 kb [15,16], its production and destruction rate will soon be in equilibrium at a neutron flux of 10^15^ n cm^-2^ s^-1^, resulting in a constant ratio of ^165^Ho to ^166m^Ho after an irradiation time of 50 d (see also Fig 2 in [17]).

Other production routes of ^166m^Ho, such as ^169^Tm(n,α)^166m^Ho or ^166^Er(n,p)^166m^Ho, are of minor importance due to the small reaction cross sections for these reactions [18,19] and the relatively well thermalized neutron flux during irradiation. For example, the fraction of epithermal and fast neutrons in the V4 beam tube of the ILL reactor is below 10%. Yet another production route of ^165^Ho proceeds via double neutron capture on ^163^Ho and largely depends on the involved thermal capture cross-sections of ^163^Ho and ^164g+m^Ho. Up to date, only the former is known as it has been recently measured to be σ_163Ho_ = (156 + 23) b for the formation of ^164g^Ho and ^164m^Ho, respectively [11]. Neutron capture reactions on ^163^Ho significantly influence the production rate and thus, the total amount of ^163^Ho after neutron irradiation. In addition, capture reactions on ^164g+m^Ho yield again ^165^Ho, a pathway that competes with both ^164g^Ho decay modes yielding ^164^Er and ^164^Dy (see hashed white lines in Fig 1). Thus, even for a chemically and isotopically pure ^162^Er material, the production of ^163^Ho in a high flux reactor is always accompanied by a number of parasitic reactions leading to ^166m^Ho.

In addition to ^166m^Ho, the irradiation will result in substantial amounts of ^170^Tm and ^171^Tm from capture reactions of ^168^Er(n,γ)^169^Er(β^-^)^169^Tm(n,γ)^170^Tm and ^170^Er(n,γ)^171^Er(β^-^)^171^Tm, respectively. Both of these isotopes are produced in the GBq range, representing the most significant hazard for handling the irradiated sample material. Moreover, any contamination of the final Ho product with kBq amounts of these isotopes will certainly deteriorate the sensitivity of ^163^Ho neutrino mass measurements. Additionally, capture reactions on impurity ^158^Dy will form ^159^Dy (t_1/2_ = 144 d). Thus, a sophisticated chemical separation of Ho not only from massive amounts of Er, but also from Tm and Dy has to be accomplished to assure a radiochemically clean product. A decrease in the production of ^166m^Ho is achieved by choosing high enrichment grades of the ^162^Er material (with low ^164^Er content) and substantially reducing the amount of impurities (Ho, Dy) present in the initial material [21]. In order to quantify the amount of ^163^Ho and parasitic ^166m^Ho formed during a reactor irradiation, a careful characterization of the initial Er material including isotope composition and impurity content should thus be performed.

Ultimately, the recovery of irradiated and purified ^162^Er is an additionally anticipated task. Due to the absence of any Er isotopes having half-lives exceeding 9.4 d (^169^Er), the recycled ^162^Er material is essentially free from any radioactivity after a cool-down period of 1 year and might be reused for new irradiations. This is especially desirable considering the price of enriched ^162^Er (> 100 $/mg). Such recycled material will moreover be depleted in ^167^Er, the strongest neutron absorber among all stable Er isotopes, which results in self-shielding and neutron flux depression during the first irradiation.

A detailed description of a possible separation procedure has been very recently given in [11]. The authors of this work suggest a pre-purification of the starting ^162^Er material in order to mitigate impurities lighter than Er. This approach maximizes the isotopic purity of ^163^Ho, but it is experimentally proven that the final Ho product will always result in a mixture of ^163^Ho, ^165^Ho and ^166m^Ho after irradiation. Apart from the described purification, the authors provide a very thorough analysis of the final ^163^Ho material yielding 1.2x10^18^ atoms of ^163^Ho, 6.3x10^17^ atoms of ^165^Ho and 7 kBq (3.8x10^14^ atoms) of ^166m^Ho from irradiating 30 mg of 20.4% enriched ^162^Er for 54 d in the ILL high flux reactor.

In parallel to results published in [11], this work describes our efforts in the frame of the HOLMES project to define and tune the production and separation process of ^163^Ho using two test batches of ^162^Er. We also report on the analysis of a final 470 mg batch of ^162^Er purchased in 2016 and give results on the separation of ^163^Ho from irradiated test batches of 2014 and 2015. In contrast to the approach presented in [11], the chemical separation procedure was based on a two-step process involving cation exchange and extraction chromatography, which has already been successfully applied for the separation of neighbouring lanthanides (see [22] for more details). It is experimentally shown that recycling of large quantities of enriched ^162^Er is feasible and might be continuously performed to satisfy needs of ^163^Ho for HOLMES. Finally, an analysis of the purified material from both test batches representing in total 1.5 mg (or 5x10^18^ atoms) of ^163^Ho will be given. No analysis of the purified ^163^Ho from the irradiated final 470 mg batch can be given since this material still awaits chemical separation.

## Materials and methods

### Materials

Three different batches of ^162^Er material have been processed at PSI between 2014 and 2016 and irradiated at ILL of which only the first two underwent a chemical purification so far. In 2014, 20.6 mg of ^162^Er_2_O_3_ (Isoflex, USA), enriched to 28.2% (^164^Er– 7.41%, ^166^Er– 32.24%, ^167^Er– 14.26%, ^168^Er– 12.26%, ^170^Er– 5.63%) and subsequently denoted as batch I, were purified from contaminants as described below and subsequently irradiated at ILL for 55 d. In 2015, 106.5 mg of ^162^Er_2_O_3_ (also Isoflex), enriched to 26.5% (^164^Er– 7.5%, ^166^Er– 31.2%, ^167^Er– 15.3%, ^168^Er– 13.0%, ^170^Er– 6.5%), were pre-purified, mixed with the previous irradiated and purified batch I and 119.5 mg of the resulting mixture (batch II) containing 26.8% ^162^Er_2_O_3_ was irradiated at ILL for 53 d. Finally, in 2016, a total mass of 544.2 mg of 25.1% enriched ^162^Er_2_O_3_ (for the isotopic composition refer to Table 1) was purchased from TraceSciences, Canada. This material, subsequently denoted as batch III, was not pre-purified and irradiated as delivered for 44 d. Each oxide from the described batches was provided to ILL in Suprasil 300 high purity quartz ampoules (Heraeus, Germany). Approximately 4 mg of batch III was kept for quantitative analysis of the material using ICP-OES, ICP-MS and neutron activation analysis (NAA).

**Table 1:** Isotopic composition and determined impurity content of ^162^Er provided by TraceScience as measured by ICP-MS, ICP-OES and NAA. The original data provided by the supplier is also given.

	Analysis PSI	TraceScience CoA
isotope	isotopic composition^1^ [%]	
Er-162	26.1(4)	25.1(14)
Er-164	6.0(2)	6.87
Er-166	30.1(7)	29.75
Er-167	15.5(4)	15.67
Er-168	15.6(6)	15.63
Er-170	6.7(2)	6.98
impurities	concentration relative to Er [at. ppm]	
Eu	15(1)^§^ ^1^^,^^3^	/
Gd	29(2)^1^	1000
Tb	7(1)^1^	/
Dy	5160(280)*^,^^2^	5400
Ho	235(25)^1^^,^ ^3^	/
Tm	360(30)^1^	900
Yb	1530(70)*^,^^2^	/
Lu	265(10)^§^ ^1^^,^^3^	/

^1^ –measured by ICP-MS

^2^ –measured by ICP-OES

^3^ –measured by NAA

^§^ - average value of two independent measurement techniques

*—the isotopic composition of Dy and Yb is non-natural, see Table 2; values denoted by “/” were not stated

### Material characterization

For a characterization of the material of batch III, a solution was prepared by dissolving 4 mg of ^162^Er_2_O_3_ in 2 mL of 1 M HNO_3_ and half of this solution used to prepare dilution series of 1:10, 1:100 and 1:1000 for ICP-OES measurements. For each dilution, 18 MΩ cm MilliQ water was provided by an in-house water purification system. Dy, Ho, Er, Tm and Yb calibration solutions in concentrations ranging from 10 ppb up to 10 ppm were prepared from their respective ICP standard solutions provided by Merck, Germany. A Perkin-Elmer Optima 3000 optical emission spectrometer was used to measure the concentration of these 5 elements in the Er stock solution. The following emission lines of the respective elements were used for analysis: Dy—396.839 nm; Ho– 345.60 nm; Er– 337.217 nm; Tm– 313.126 nm; Yb– 328.937 nm.

Another 400 μL of the stock solution was evaporated in a PE vessel and irradiated for 10^4^ s in the neutron activation facility of the PSI spallation neutron source (SINQ). 3.9 mg of IRMM-527 (Al-0.1%Co) alloy, Sigma Aldrich, USA, was used as flux monitor. Gamma spectra of the ^162^Er sample were recorded 5h, 24h and 4d after end of irradiation. Then the sample was irradiated with neutrons for another 10^4^ s and immediately underwent chemical separation by ion exchange chromatography as described below. The separation was monitored by γ‑spectrometry using a coaxial p-type HPGe detector, Mirion Technologies, USA. The Genie2000 software, Canberra, was used to evaluate the recorded spectra. Each sample was measured for at least 10 min to provide a statistical uncertainty below 1% for the peak area of interest. The separated fractions of Dy, Er and Yb were then measured for their isotopic composition using a sector-field based mass spectrometer Element 2, Thermo Fischer Scientific, Germany. The ICP mass spectrometer was operated in low resolution mode and wet plasma conditions. All measurement solutions were prepared from high purity nitric acid and water in polymer vessels.

### Material separation

For the separation of ^163^Ho from irradiated ^162^Er of each test batch I and II, the ampoule containing the oxide was cracked, the material dissolved in 5 mL of 7 M HNO_3_ and the pH adjusted to 4 with approximately 10 mL NH_4_OH. Approx. 0.5 mg of ^162^Er activated at the PSI SINQ facility to yield ^171^Er (t_1/2_ = 7.52 h) was added prior to each separation to be able to monitor the elution of Er using γ‑spectrometry. The solution was then loaded onto a column (L = 23 cm, d = 1 cm) containing 19 g of the cation exchange resin Aminex HPX87H from BioRad Laboratories, USA. Batch I containing 20.6 mg of ^162^Er_2_O_3_ was entirely loaded on the column, while the solution of batch II containing 119.5 mg of ^162^Er_2_O_3_ was divided in half to allow two independent separations. The elution of the lanthanides was performed at room temperature using increasing concentrations of α-hydroxy-isobutyric acid (HIBA) in a similar way as described in [22] via a peristaltic pump ISM834C (Ismatec, Switzerland). The gradual elution of the lanthanides was performed in 10 mL steps using a flow rate of 0.66 mL/min and was monitored by γ‑spectrometry. All fractions containing chemically pure Ho were unified, the resulting 30 mL of solution acidified with 6 mL 1 M HNO_3_ and loaded on a column (L = 16 m, d = 1 cm) containing 4.7 g of LN1 resin (Triskem, France) for final purification from HIBA and residual contaminants using increasing concentrations of HNO_3_. Fractions containing Er were unified to yield 70 mL of solution, acidified with 15 mL 1 M HNO_3_ and similarly loaded on LN1 resin for HIBA stripping. After the elution of Er with 4 M HNO_3_, the acid is evaporated and the residue burned to the oxide in a quartz ampule (Heraeus, Germany) using a flame torch. The total recovery of recycled ^162^Er is determined gravimetrically using an AE 100 balance, Mettler-Toledo, Switzerland.

The purified Ho fractions were unified, evaporated to dryness and redissolved in 10 mL of 1 M HNO_3_. This solution was subsequently investigated with ICP-MS and γ‑spectrometry to determine the content of ^163^Ho, ^165^Ho and ^166m^Ho. The concentration of ^163^Ho and ^165^Ho was measured by dilution series using a Ho standard solution, Merck, Germany, as reference. All uncertainties are reported according to the “guide to the expression of uncertainty in measurement” (GUM) with a coverage factor of k = 1.

## Results and discussion

### Material composition

The results of the ICP-MS, ICP-OES and neutron activation analysis of batch III is given in Table 1 together with the data from the original Certificate of Analysis provided by the supplier. The results obtained from all three analytical methods agree well within the statistical uncertainties of the measurement. The only significant deviation was found between results obtained by NAA and ICP-MS on Dy and Yb, since both of these impurity elements were found to have a non-natural composition.

The determined isotopic composition of Er tends to agree with the numbers provided by the supplier. It should be noted that the impurity content given by the supplier partially deviates from what was found by the analysis at PSI. This fact is due to isobaric interferences arising from Dy and Yb impurities, which are present in the material in the ‰ range. During the isotope enrichment process, not only the main product ${}_{Z}^{A}N$ is enriched, but also its isobaric impurities such as ${}_{Z-1}^{\;A}N$ or ${}_{Z+1}^{\;A}N$. Thus, in isotopically enriched samples of ^162^Er, the Dy impurity gets enriched in ^162^Dy. This fact has been confirmed by ICP-MS measurements of the Dy isotopic composition given in Table 2. Apart from much lower Gd content, the analysed sample also contained a significant amount (1530 ppm) of Yb enriched in ^176^Yb, which has not been reported in the original analysis provided by the supplier. The Yb impurity is believed to originate from previous enrichment runs of ^176^Yb operated at the same enrichment facility.

**Table 2:** The isotopic composition of Dy and Yb present as impurities in the analysed ^162^Er batch III; measured by ICP-MS.

isotope	abundance	isotope	abundance
Dy-156	0.02%	Yb-168	0.16%
Dy-158	0.03%	Yb-170	2.17%
Dy-160	0.35%	Yb-171	6.03%
Dy-161	8.40%	Yb-172	8.84%
Dy-162	64.13%	Yb-173	10.42%
Dy-163	15.84%	Yb-174	15.08%
Dy-164	11.23%	Yb-176	57.31%

From the relative abundance of ^164^Er and the concentration of the Dy and Ho impurities in the initial batch III material it is possible to deduce their expected contribution towards the ^166m^Ho production according to Fig 2. As calculations show, roughly 80% of parasitic ^166m^Ho will be formed from ^164^Er, while 8% is expected to be produced from ^164^Dy. The initial content of 235 ppm Ho as well as ^163^Dy and ^162^Dy almost equally contribute with 4% to the total ^166m^Ho inventory. Assuming a ^163^Ho burn-up as given in [11], the expected ^163^Ho:^166m^Ho atomic ratio will be in the order of 3x10^3^ according to calculations.

### ^163^Ho separation

**Fig 3:**
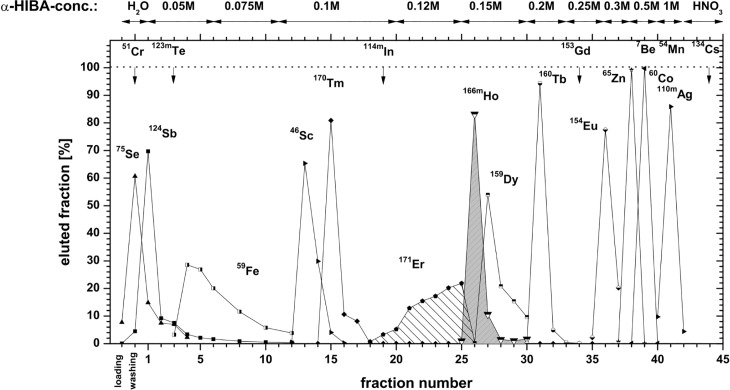
Separation profile of Ho from irradiated ^162^Er of batch II using exchange chromatography on Aminex HPX87H resin with increasing concentrations of α-hydroxy-isobutyric acid.

**Fig 4:**
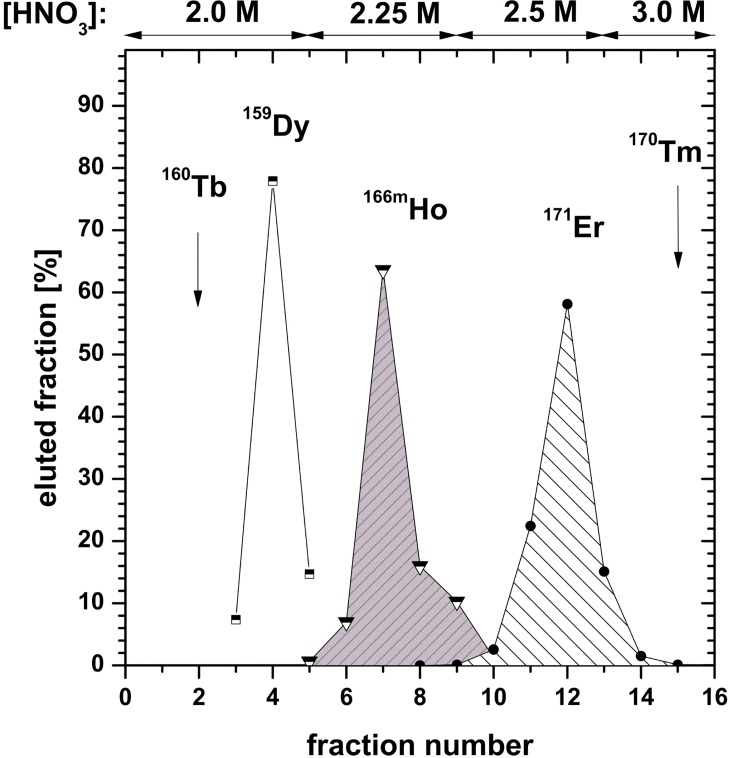
Separation profile of the final Ho purification on the LN1 resin using increasing concentrations of nitric acid.

The separation profile of the batch II material irradiated for 53 d in 2015 is given in Fig 3. As it can be seen from Fig 3, a large number of radioisotopes is produced during the irradiation for 53 d at the ILL high flux reactor. Most elements may be efficiently separated on a cation exchange resin from the Ho product, although some overlap with Er and Dy was observed. This is due to the fact of high column loading that leads to inferior separation of the lanthanides [23]. ^170/171^Tm, ^154^Eu, ^65^Zn and ^60^Co, representing isotopes with the highest contribution to activity and dose rate, are efficiently separated. The absence of their γ-lines in the final Ho product yields decontamination factors exceeding 10^6^. The additional purification of the Ho product on the LN1 resin even further decreased the impurity contribution from Er and Dy. The corresponding separation profile is given in Fig 4.

The final mass of isolated ^163^Ho, the measured activity of ^166m^Ho and the impurity content originating from the batches I and II are given in Table 3. The cumulative amount of ^163^Ho produced during these runs is approximately 1.5 mg, which represents roughly 27 MBq or 5x10^18^ atoms of this isotope. The contribution of signals at m/q = {161–170} relative to m/q = 163 is assigned to the sum of the respective isobars. Conservatively assuming that all signals at m/q = 166 are originating from ^166^Er, the overall separation factor Ho/Er is calculated to be at least 10^5^ and 3x10^4^ for batches I and II, respectively. The cumulative separation factor for Ho/Dy after both chromatographic separations is difficult to assess, since signals at m/q = {162–164} have isobaric interferences from ^162^Er, ^163^Ho and ^164^Er. The only Dy isotope free from isobaric interferences is ^161^Dy, of which the abundance, however, was not measured. A more sophisticated analysis of the final product using resonant ionization mass spectrometry in connection with NAA as described in [11] would resolve the issue. In any case it can be stated with certainty that both ^163^Ho samples processed so far contain less than 1% of (Dy+Er). This indicates that the separation procedure presented in this work yields ^163^Ho of satisfactory quality similar to results obtained in [11].

**Table 3:** Overview on ^163^Ho samples with their respective mass and composition.

batch number	m(^163^Ho) [mg]^1^	A(^163^Ho) [MBq]	m(^165^Ho) [mg]^1^	^163/165^Ho at. ratio^1^	A(^166m^Ho) [kBq]^2^	^163/166m^Ho at. ratio^1^^,^^2^
I	0.25(1)^3^	4.4	0.14(1)	1.83	6.3(1)	2.9x10^3^
II	1.28(3)^4^	22.7	0.84(2)	1.52	37.7(3)	2.4x10^3^
III	6*	100*	4*	1.5*	200*	2x10^3^*

^1^ –measured by ICP-MS

^2^ –measured by γ‑spectrometry

^3^ –impurity content: {161,162,164,166,167,168,169,170}/163 < 0.0005

^4^ –impurity content: 162/163–0.00125; 164/163–0.0025; 166/163–0.0011; 168/163–0.0006

*—estimated numbers calculated using the ChainSolver program

Regarding the overall separation yield a clear dependence on the total mass of ^162^Er is noteworthy. While for a separation of ^163^Ho from 20.6 mg Er_2_O_3_ (batch I) a total yield of 98.4% was achieved, the yield dropped down to 79.2% in case of the batch II material where 119.5 mg of Er_2_O_3_ were processed. This is due to some unwanted overlapping of Er/Ho/Dy elution peaks as seen in Fig 3 as a result of higher column loadings with respect to the previous batch. In order to mitigate this problem in future, it is advisable to partition the column separation step into several runs. The recovery of ^162^Er was successful in every separation and more than 90% of the material could be recycled and used for new irradiations.

As can be seen from numbers given in Table 3, the production of ^163^Ho scales linearly with the amount of ^162^Er irradiated for ≈50 days at the ILL reactor. Due to higher ^162^Er enrichment of batch I, the total ^163/165^Ho atomic ratio is consequently higher. Enrichments above 90% of ^162^Er or much shorter irradiation times would be needed in order to make this ratio exceed 6. It is interesting to note that according to the measured data given in Table 3, the atomic ratio of ^165^Ho to ^166m^Ho is constant through batch I and II and equals roughly 1.6x10^3^. This is due to a dynamic equilibrium reached after 50 d of irradiation in the production and destruction pathways leading to ^165^Ho and ^166m^Ho, as has been mentioned earlier.

The 544.2 mg of enriched ^162^Er_2_O_3_ of batch III as well as 110.1 mg of recovered ^162^Er_2_O_3_ from batch II were irradiated at the high flux reactor at ILL in 2017 and will undergo chemical separation soon. The separated material is intended for neutron cross section measurements at the CERN n_TOF facility prior to final delivery to the HOLMES isotope separator. With a total amount of 6 mg ^163^Ho, this new irradiated of batch III will fully cover the needs of this isotope within the frame of the HOLMES project [5].

## Conclusions

We have successfully separated 1.5 mg of ^163^Ho, corresponding to 27 MBq, from macro amounts of enriched ^162^Er. This amount of ^163^Ho is at least one order of magnitude higher than can be reasonably achieved by any other production route involving e.g. proton irradiations of Dy targets [10]. It was also shown that a recycling of ^162^Er is easily achievable to meet with ^163^Ho requirements for nuclear physics experiments devoted to measure the mass of the neutrino and to deduce the ^163^Ho neutron capture cross section at stellar neutron energies. However, this production route comes with the disadvantage of parasitic formation of ^166m^Ho, which cannot be separated from the wanted ^163^Ho by chemical means. A sophisticated, highly efficient and selective mass separation has to be performed for reliable removal of the isotopic contaminant in order to allow clean calorimetric measurements of the ^163^Ho electron capture decay. A mass separation involving laser resonant ionization has already been successfully proven to reduce the ^166m^Ho amount by three orders of magnitude [21].
